# Antecedent occipital alpha band activity predicts the impact of oculomotor events in perceptual switching

**DOI:** 10.3389/fnsys.2013.00019

**Published:** 2013-05-24

**Authors:** Hironori Nakatani, Cees van Leeuwen

**Affiliations:** ^1^Okanoya Emotional Information Project, Exploratory Research for Advanced Technology, Japan Science and Technology AgencyWako-shi, Japan; ^2^Emotional Information Joint Research Laboratory, RIKEN Brain Science InstituteWako-shi, Japan; ^3^Laboratory for Perceptual Dynamics, Experimental Psychology Unit, Faculty of Psychological and Educational SciencesKU Leuven, Leuven, Belgium

**Keywords:** ambiguous figures, Necker cube, perception, electroencephalogram (EEG), blinking, saccade

## Abstract

Oculomotor events such as blinks and saccades transiently interrupt the visual input and, even though this mostly goes undetected, these brief interruptions could still influence the percept. In particular, both blinking and saccades facilitate switching in ambiguous figures such as the Necker cube. To investigate the neural state antecedent to these oculomotor events during the perception of an ambiguous figure, we measured the human scalp electroencephalogram (EEG). When blinking led to perceptual switching, antecedent occipital alpha band activity exhibited a transient increase in amplitude. When a saccade led to switching, a series of transient increases and decreases in amplitude was observed in the antecedent occipital alpha band activity. Our results suggest that the state of occipital alpha band activity predicts the impact of oculomotor events on the percept.

## Introduction

Oculomotor behavior such as blinking and saccades are prominent in visual perception. We spontaneously blink our eyes every few seconds. The role of blinking goes beyond merely moistening the eyes; among other things, blinking reflects the deployment of attentional resources. For example, blinking frequency decreases when cognitive demand increases (Veltman and Gaillard, 1998). Also, blinking tends to occur at breakpoints of attention (Nakano et al., 2009; Nakano and Kitazawa, 2010) and may have an active role in the disengagement of attention (Nakano et al., 2013).

Saccadic eye movements occur with a similar intensity; several times per second we spontaneously shift our gaze from one location to the next. Saccades are closely associated with visual attention. For example, the allocation of spatial attention is tightly time-locked to saccade execution (Filali-Sadouk et al., 2010). Neuroimaging studies have shown that attention and saccade planning share common neural substrates in the frontal and parietal areas (Corbetta et al., 1998; Nobre et al., 2000). Attention modulates the content of our percept; when a certain part within the visual input is selectively attended, the corresponding information is enhanced (Chelazzi et al., 2001).

Although both blinking and saccades are tightly associated with attentional processes and cause transient changes in retinal stimulation, we are seldomly aware of this. Sensitivity to visual input normally is actively suppressed during blinking and saccades (Volkmann et al., 1980; Burr et al., 1994; Bristow et al., 2005). The blink and saccadic suppression mechanisms, in combination with the constructive abilities of perception (Koenderink et al., 2012), explain why our visual experience remains continuous across transient interruption of visual input by blinking.

However, recent studies suggested that both blinking and saccades could have an impact on our visual experience. Blinking could, for instance, trigger illusory motion in the Rotating Snakes illusion (Otero-Millan et al., 2012). These authors showed that, besides blinking, also microsaccades led to the perception of illusory motion. Together, these results indicate that oculomotor events sometimes lead to visual transients affecting the percept. Other such effects have been reported in the case of multistable perception. For example, in one of our previous studies, we found that some, but not all, blinking and saccades led to *perceptual switching* in an ambiguous figure (Nakatani et al., 2011). Perceptual switching is the phenomenon that a percept switches spontaneously between possible interpretations of an ambiguous figure (e.g., Attneave, 1971; Leopold and Logothetis, 1999; Ito et al., 2003; Parker and Krug, 2003; Nakatani and van Leeuwen, 2005, 2006; Nakatani et al., 2011, 2012).

Why is it that some oculomotor events lead to changes in our visual percept, whereas others do not? We propose that the ones that lead to changes are preceded by a shift in visual attention. Attention modulates the content of our percept by selectively enhancing an attended part within the visual input (Chelazzi et al., 2001). We, therefore, expect that attention-related brain signals predict whether oculomotor events have an influence on the percept. With new analyses on previously reported data about oculomotor behavior and the electroencephalogram (EEG) in perceptual switching (Nakatani et al., 2011), we show that preceding occipital alpha band activity predicts the impact on the percept of blinks or saccades.

## Materials and methods

### Participants

Six participants (aged 21–34 years) participated in this study. Participants gave their written informed consent to the study. The Research Ethics Committee of the RIKEN had approved our procedures.

### Experimental design

**Figure 1:**
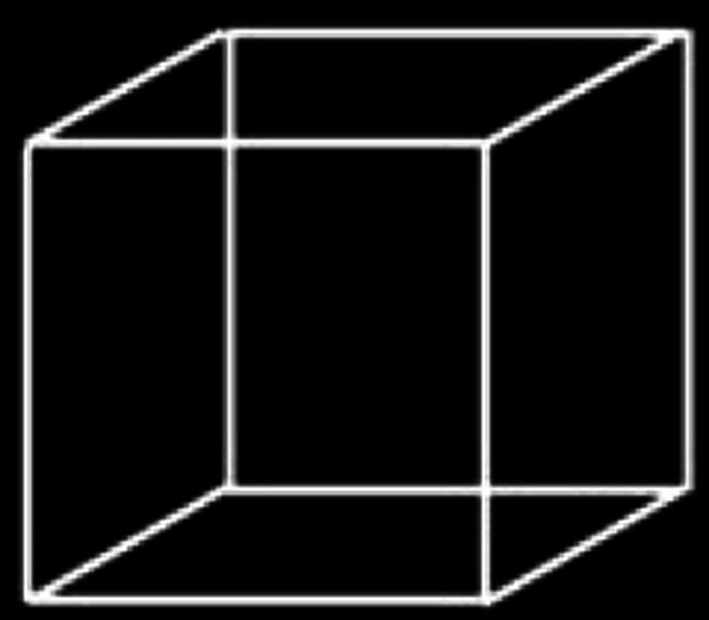
**Necker cube used as visual stimulus in our experiment.** The stimulus was presented as a white line-drawing on a black ground. The visual angle of presentation was 5.0°. The stimulus was continuously presented for 240 s.

Since some of the results from the present study have been published earlier; here we report only the main characteristics of the design. For further details, see Nakatani et al. (2011). The experiment consisted of two conditions: a *perceptual switching* condition and a *stimulus initiated* condition (Nakatani et al., 2011). Each lasted 240 s and both conditions were presented in counterbalanced order within a session. In the *perceptual switching* condition, a Necker cube (Figure 1) was continuously presented as a white line-drawing, subtending 5° of visual angle, on a black ground. The stimulus was shown at eye-height in a sound proof room with reduced ceiling illumination. Since perceptual switching may fail to occur if participants know only one of the possible interpretations of an ambiguous figure (Rock et al., 1994), in order to assure that all participants started with equal information, they were advised before the experiment that the Necker cube could be seen in two alternative orientations, which are referred to here as “downward” and “upward” orientation. Participants pressed a response button corresponding to the perceived switching direction, i.e., from upward to downward or vice versa. They had been instructed to do so whenever their visual percept of the Necker cube reversed but not when it merely became inconsistent or vague. The *stimulus initiated* condition was a control condition, which is not relevant for the present paper. Three sessions separated by a break were conducted for each participant.

### Measurements of oculomotor events and EEG

Oculomotor events (blinks and saccades) were measured with an SR Research Eyelink system in three participants and an SR Research Eyelink 1000 system in the others. Both are video-based eye-tracking systems. As the SR Research Eyelink system was broken halfway during our study, we used the SR Research Eyelink 1000 system for the remaining participants. We presented the stimulus to both eyes, and measured oculomotor events (in particular, blinks and saccades) from the dominant eye.

Simultaneously with oculomotor events, we also measured EEG. Disk-type Ag/AgCl electrodes were placed on O1, O2, P3, Pz, P4, F3, Fz, and F4 recording sites in accordance with the international 10/20 system (Jasper, 1958). When the SR Research Eyelink 1000 system was used for oculomotor events measurement, it was possible to place additional electrodes on C3, Cz, and C4 recording sites. Reference and ground electrodes were placed on left and right ears of each participant, respectively. Vertical and horizontal electrooculogram (EOG) were also recorded. Sampling frequency was 500 Hz.

### EEG analysis

We used independent component analysis (Hyvärinen and Oja, 2000) to reduce oculomotor artifacts in EEG recordings. Using the FastICA algorithm (Hyvärinen and Oja, 1997), we decomposed the EEG and EOG recordings into independent components. We then reconstructed the EEG recordings after we removed components that had larger correlation with vertical or horizontal EOG than with EEG.

In order to analyze EEG in the time-frequency domain, we applied a continuous wavelet transform to EEG. The mother function of the wavelet transform was the complex Gabor function *g*(*t*),

$$g(t)=\frac{1}{2\sqrt{\pi \alpha }}\exp\left(-\frac{t^{2}}{4\alpha^{2}}\right)\exp\left(i2\pi t\right),$$

where α = 0.5. The size of the mother function was about 5 cycles. Wavelet coefficients of a signal *x*(*t*), each channel of EEG, were obtained as follows:

$$W\left(t,f\right)=\sqrt{f}\int x\left(t\right)g^{*}\left(f\left(\tau -t\right)\right)d\tau ,$$

where *g*(*t*)^*^ is the complex conjugate of a complex Gabor function, and *t* and *f* indicate time and frequency, respectively. We then obtained EEG amplitude |*W* (*t, f*)| in the time-frequency domain.

After the continuous wavelet transform, we calculated average waveforms of EEG amplitude that were aligned with oculomotor events of interest for each participant. We used trial average data per participant for the following statistical analyses.

**Figure 2:**
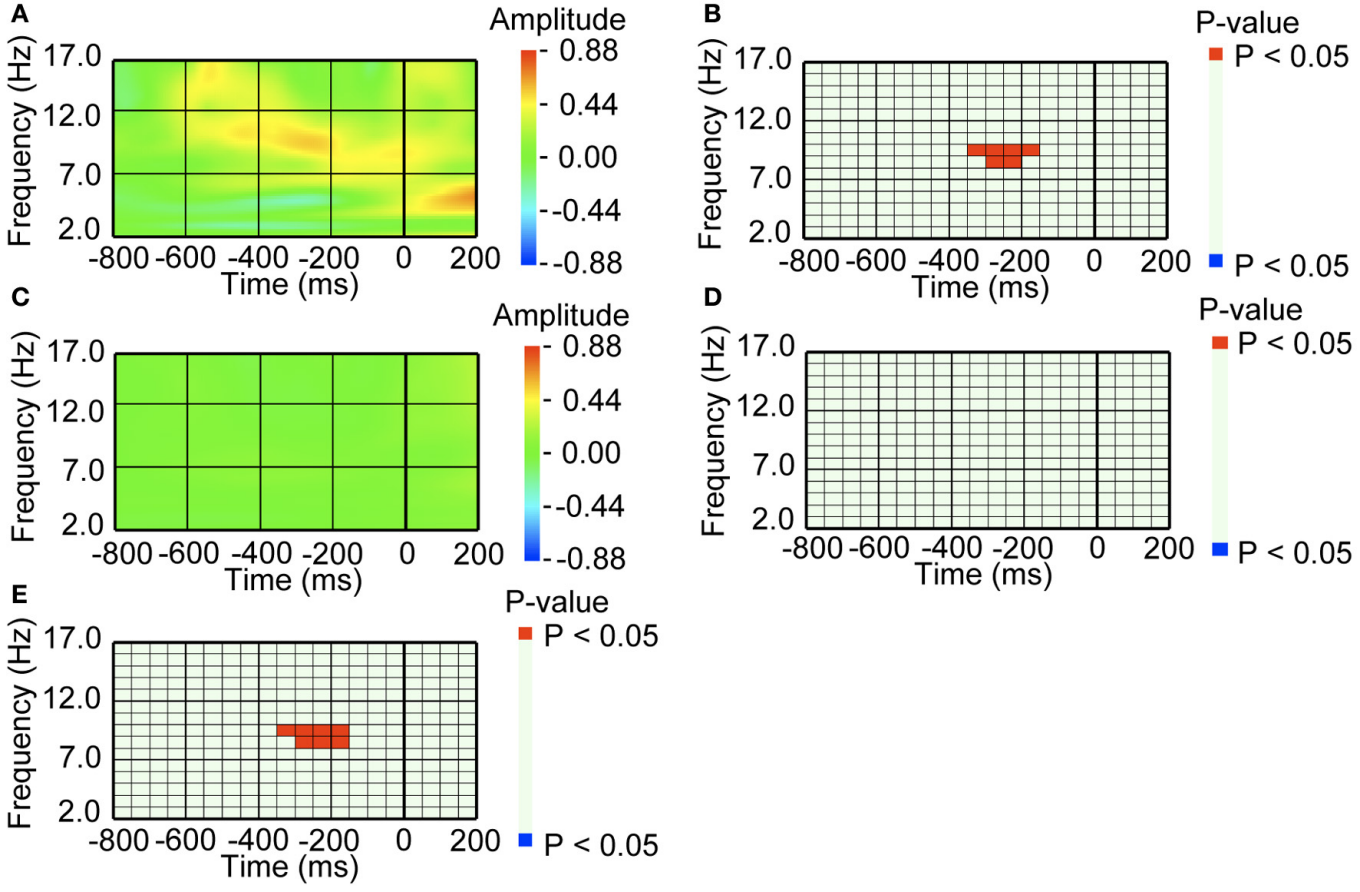
**An amplitude increase in occipital alpha band activity preceded blinking, when blinking was followed by perceptual switching. (A)** Average waveform of the occipital recordings (O2) aligned with the *pre-switch* blinkings that were followed by perceptual switching. The amplitude was Z-transformed, and the amplitude *zero* indicates baseline amplitude. **(B)** Statistical difference between average waveform aligned with the *pre-switch* blinkings and baseline amplitude. The colors *red* and *blue* denote that amplitude was larger in average waveform or baseline amplitude, respectively (*P* < 0.05). **(C)** Average waveform of the occipital recordings (O2) aligned with the *no-switch* blinkings that were not followed by perceptual switching. The amplitude was Z-transformed, and the amplitude *zero* indicates baseline amplitude. **(D)** Statistical difference between average waveform aligned with the *no-switch* blinkings and baseline amplitude. The colors *red* and *blue* denote that amplitude was larger in average waveform or baseline amplitude, respectively (*P* < 0.05). **(E)** Statistical difference between average waveform aligned with *pre-switch* blinks and average waveform aligned with the *no-switch* blinks. The colors *red* and *blue* denote that amplitude was larger in the *pre-switch* blinking-related average waveform or the *no-switch* blinking-related average waveform, respectively (*P* < 0.05).

To detect EEG episodes that were associated with oculomotor events preceding perceptual switching (*pre-switch* oculomotor events), we conducted two types of comparisons. First, we compared average waveforms of EEG amplitude with a baseline amplitude. We defined a separate baseline amplitude for each frequency. The baseline amplitude was the mean amplitude at each frequency calculated from the entire 12 min of EEG recorded from the three experimental sessions. Second, we compared average waveforms of EEG amplitude aligned with *pre-switch* oculomotor events with average waveforms of EEG amplitude aligned with *no-switch* oculomotor events. For statistical comparison, we applied the bootstrap resampling method (see below in this section) to the EEG activity that preceded the oculomotor events. To avoid massive multiple comparison, we divided the time-frequency domain into relatively large segments. The size of each segment was 50 ms in width and 1 Hz in height (see Figure 2B) and the amplitude of each segment was defined by the mean amplitude within the segment. When a certain EEG episode was statistically different in amplitude of the average waveform compared to the baseline, we considered that this episode was associated with the oculomotor event of interest. Furthermore, we considered that the episode occurred in relation to a *pre-switch* oculomotor event when its average waveform in pre-switch oculomoter events was statistically different from that in *no-switch* oculomotor events.

For graphic representation of average waveforms in the Results section, we used Z-transformed amplitude. We used mean and standard deviation of EEG amplitude calculated from 12 min of whole recordings for Z-transform. As baseline amplitude used for statistical testing was 0 in the Z-transformed amplitude, this makes it easier to observe whether EEG amplitude increased or decreased in relation to oculomotor events of interest. We applied Z-transform to un-averaged EEG amplitude |*W* (*t, f*)|. We calculated average waveforms of Z-transformed amplitude that were aligned with oculomotor events of interest for each participant, and then calculated average waveforms across participants. We graphed the average waveforms across participants.

We applied the bootstrap resampling method (Efron, 1979; Efron and Tibshirani, 1986), in order to evaluate the statistical significance of differences in parameters of interest between two data sets (Nakatani et al., 2011). This method is a non-parametric approach and, therefore, there is no need to assume that the parameters of interest follow the normal distribution. When we compare two data sets *x*^*i*^_*A*_ and *x*^*i*^_*B*_, we first calculate the difference between them as follows:

$$x_{\text{diff}}^{i}=x_{A}^{i}-x_{B}^{i},$$

where *i* is the integer number that takes from 1 to *n* to identify individual participants, and *n* is the number of participants (*n* = 6, in this study). Then, we calculated group average of *x*^*i*^_diff_. That is,

$${\bar{x}}_{\text{diff}}=\frac{1}{n}\sum_{i=1}^{n}x_{\text{diff}}^{i}.$$

The null hypothesis to be tested is that there is no difference between the two data sets. That is,

$${\bar{x}}_{\text{diff}}=\text{0}.$$

We generated bootstrap parameters for ${\bar{x}}_{\text{diff}}$ that satisfy the null hypothesis. The distribution of these bootstrap parameters is used to evaluate the statistical significance of ${\bar{x}}_{\text{diff}}$. We first generated the bootstrap parameters for each participant,

$$x_{\text{diff}}^{*i}=\left(x_{A}^{i}-\bar{x_{A}}\right)-\left(x_{B}^{i}-\bar{x_{B}}\right).$$

As the group average of x^*i^_diff_, ${\bar{x}}_{\text{diff}}^{*}$ is 0, the bootstrap parameters satisfy the null hypothesis. With the bootstrap resampling method (Efron, 1979; Efron and Tibshirani, 1986), we estimated the distribution of ${\bar{x}}_{\text{diff}}^{*}$, that can be considered as the distribution of ${\bar{x}}_{\text{diff}}$ in case it satisfies the null hypothesis, and obtained a Monte Carlo approximation of the *p*-value for ${\bar{x}}_{\text{diff}}$.

To deal with the multiple comparison problem, we used a cluster-based permutation test (Bullmore et al., 1999; Maris and Oostenveld, 2007; Groppe et al., 2011). This method is a non-parametric approach, suitable to detect broadly distributed effects (Groppe et al., 2011). We ignored all segments of the time-frequency domain of which the test statistic, ${\bar{x}}_{\text{diff}}$, does not exceed a pre-determined threshold, equaling an uncorrected *p*-value of 1%. The remaining segments were composed into clusters by grouping together adjacent segments on the time-frequency domain. We calculated the mean value of test statistic for each cluster, in order to define a cluster-level value for the test statistic. The most extreme cluster-level value of the test statistic was used for permutation procedures, in order to derive a distribution for the null hypothesis. Same as in statistical testing with the bootstrap resampling method, the null hypothesis was: zero difference between the two data sets. The corrected *p-value* of each cluster was derived from its ranking in the null hypothesis distribution, and then each segment of the cluster was assigned the *p*-value of the entire cluster. We considered significant those segments of which the corrected *p*-value exceeded the 5% level. From hereon, corrected significance levels only will be reported.

For our analyses, we used custom scripts written in C (gcc compiler version 4.2.1 on MacOSX 10.6.8).

### Temporal distributions of saccadic probabilities

To investigate the relationship between blinking and saccades, we calculated temporal distributions of saccadic probabilities in alignment to onsets of blinking of interest. Choosing onsets of blinking of interest as the reference (0 ms), if certain types of saccades are time-locked to these blinks, this would be revealed by a peak in the aligned saccade frequency temporal distribution. Likewise, if certain types of saccades are systematically omitted in a time-locked fashion, the distribution would show a dip. We calculated the occurrence probability of saccades within 100 ms width bin with 50 ms overlap and obtained average probabilities across participants.

## Results

In our previous study (Nakatani et al., 2011), about 5% of blinking occurred in a short period in which blinking was temporarily increased, about 1000 ms prior to a switching response. Such *pre-switch* blinks were followed by a transient amplitude increase of theta band activity. The switches showed a larger than average bias to the interpretation of the Necker cube that individual participants preferred. About 150 ms prior to a switching response, ~5% of saccades occurred in a short period in which saccade frequency was temporarily increased. Such *pre-switch* saccades were preceded by a transient amplitude increase of theta band activity. The direction of the saccades was systematically related to the interpretation of the Necker cube after the switch. For the blinks and saccades occurring in these two specific intervals, we here investigated the EEG activity prior to these events.

For the *pre-switch* blinking, alpha band activity (around 10 Hz) over the occipital area exhibited an increase in amplitude around 250 ms before the onset of the blink (Figure 2A). The increase was significant at *P* < 0.05 level, compared to baseline amplitude (Figure 2B). In the same intervals prior to *no-switch* blinks, i.e., blinks that were not followed by perceptual switching, the occipital area did not exhibit such amplitude increase (Figures 2C,D). We also directly compared the *pre-switch* blinking-related EEG with the *no-switch* blinking-related EEG, and found that the amplitude increase was specific to the *pre-switch* blink-related EEG (Figure 2E).

**Figure 3:**
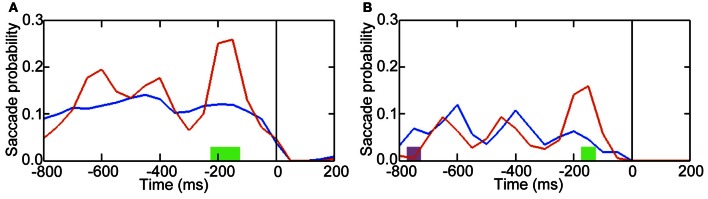
**Probabilities of preceding saccades transiently increased when blinking led to perceptual switching.** Time 0 indicates onset of blinking. **(A)** The color *red* denotes saccade probabilities when blinking led to a switch response (button press) approx. 1000 ms after blinking. *Blue* denotes saccade probabilities when blinking did not lead to a switch response. *Green* denotes periods that exhibited increased saccade probabilities when blinking led to a switch response (*P* < 0.05). **(B)** The color *red* denotes the probabilities of the *pre-blink* saccades whose directions were consistent with the preferred interpretation of the Necker cube after the switch. *Blue* denotes the probabilities of the *pre-blink* saccades whose directions were inconsistent with the preferred interpretation. *Green* denotes periods that exhibited increased probabilities of the *pre-blink* saccades whose directions were consistent with the preferred interpretation (*P* < 0.05). *Purple* denotes periods that exhibited increased probabilities of the *pre-blink* saccades whose directions were inconsistent with the preferred interpretation (*P* < 0.05).

To investigate whether the amplitude increase over occipital areas was due to an eye-movement artifact, we compared saccade probabilities between *pre-switch* and *no-switch* blinks (Figure 3A). Around the time when the occipital area exhibited an amplitude increase in relation to *pre-switch* blinks, no difference in saccade probabilities between *pre-switch* and *no-switch* blinks was observed (*P* = 0.740 > 0.05, for the 300–250 ms interval prior to blinking onset). Saccade probabilities were significantly larger, however, in the interval between the occipital alpha band activity and blinking onset in *pre-switch* blinks, compared with *no-switch* blinks (*P* = 0.048 < 0.05 for the 200–150 ms prior to blinking).

These *pre-blink* saccades potentially constitute an even earlier oculomotor predictor of an ensuing switch than the subsequent blink. As they preceded *pre-switch* blinking that occurred 1000 ms before switch responses, they clearly differ in their timing from the *pre-switch* saccades currently under investigation. In our previous study (Nakatani et al., 2011) *leftward* (130–240°) *pre-switch* saccades tended to be followed by perceptual switching to a downward interpretation of the Necker; switching to an upward interpretation tended to follow *rightward* (300–60°) *pre-switch* saccades (in a polar coordinate system with right = 0°, top = 90°, left = 180°, bottom = 270°). If the present, earlier *pre-blink* saccades reflect the switching process, we may expect that the same relationship holds; the direction of the *pre-blink* saccades was expected to be associated with the preferred interpretation of the Necker cube after a switch, because the *pre-switch* blinks led to perceptual switching to the preferred interpretation (Nakatani et al., 2011). Downward interpretation was the preferred one for five out of six participants and upward interpretation was the preferred one for other one participant (Nakatani et al., 2011). As shown in Figure 3B, indeed this relationship was evident in our data. We may conclude that these *pre-blink* saccades reflect the ensuing switch in a manner similar to the *pre-switch* saccades.

**Figure 4:**
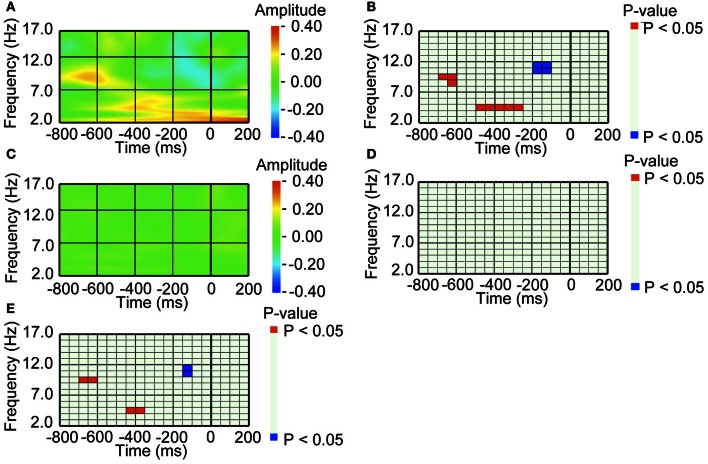
**A series of amplitude increase and decrease in occipital alpha band activity preceded saccade, when saccade was followed by perceptual switching. (A)** Average waveform of the occipital recordings (O2) aligned with the *pre-switch* saccades that were followed by perceptual switching. The amplitude was Z-transformed, and the amplitude *zero* indicates baseline amplitude. **(B)** Statistical difference between average waveform aligned with the *pre-switch* saccades and baseline amplitude. The colors *red* and *blue* denote that amplitude was larger in average waveform or baseline amplitude, respectively (*P* < 0.05). **(C)** Average waveform of the occipital recordings (O2) aligned with the *no-switch* saccades that were not followed by perceptual switching. The amplitude was Z-transformed, and the amplitude *zero* indicates baseline amplitude. **(D)** Statistical difference between average waveform aligned with the *no-switch* saccades and baseline amplitude. The *colors red* and *blue* denote that amplitude was larger in average waveform or baseline amplitude, respectively (*P* < 0.05). **(E)** Statistical difference between average waveform aligned with *pre-switch* saccades and average waveform aligned with the *no-switch* saccades. The colors *red* and *blue* denote that amplitude was larger in the *pre-switch* saccade-related average waveform or the *no-switch* saccade-related average waveform, respectively (*P* < 0.05).

We next analyzed the EEG activity before the *pre-switch* saccades, that occurred 150 ms before switch responses (Nakatani et al., 2011). For the *pre-switch* saccades, the occipital area exhibited an increase in the alpha band (around 10 Hz) 650 ms before and a decrease in the higher alpha band (around 11 Hz) 150 ms before the onset of the saccade, compared to baseline amplitude (Figures 4A,B). The lower theta band (around 5 Hz) activity before 400 ms also exhibited increased amplitude, as we had observed in our previous study (Nakatani et al., 2011). In contrast, for the *no-switch* saccades, the occipital area did not exhibit such amplitude in- and decrease (Figures 4C,D). We also directly compared the *pre-switch* saccade-related EEG with the *no-switch* saccade-related EEG, and found that the amplitude in- and decrease were specific to the *pre-switch* saccade-related EEG (Figure 4E).

**Figure A1:**
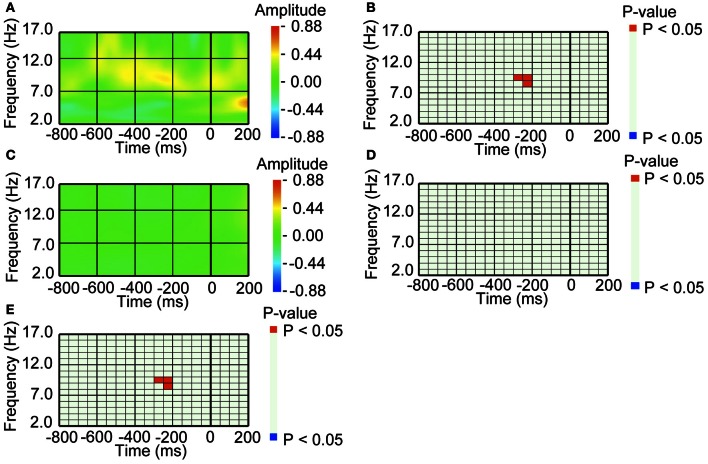
**EEG amplitude preceding blinking, when the mother wavelet with a size of 3 cycles was used for the continuous wavelet transform. (A)** Average waveform of the occipital recordings (O2) aligned with the *pre-switch* blinks. Amplitude was Z-transformed, and the amplitude *zero* constitutes the baseline. **(B)** Statistical difference between average waveform aligned with the *pre-switch* blinks and baseline amplitude. The colors *red* and *blue* denote that amplitude was larger in the average waveform or baseline amplitude, respectively (*P* < 0.05). **(C)** Average waveform of the occipital recordings (O2) aligned with the *no-switch* blinks. The amplitude was Z-transformed, and *zero* indicates baseline amplitude. **(D)** Statistical difference between average waveform aligned with the *no-switch* blinks and baseline amplitude. The colors *red* and *blue* denote that amplitude was larger in average waveform or baseline amplitude, respectively (*P* < 0.05). **(E)** Statistical difference between the average waveform aligned with the *pre-switch* blinks and the average waveform aligned with the *no-switch* blinks. The colors *red* and *blue* denote that amplitude was larger in the *pre-switch* blinking-related average waveform or the *no-switch* blinking-related average waveform, respectively (*P* < 0.05).

**Figure A2:**
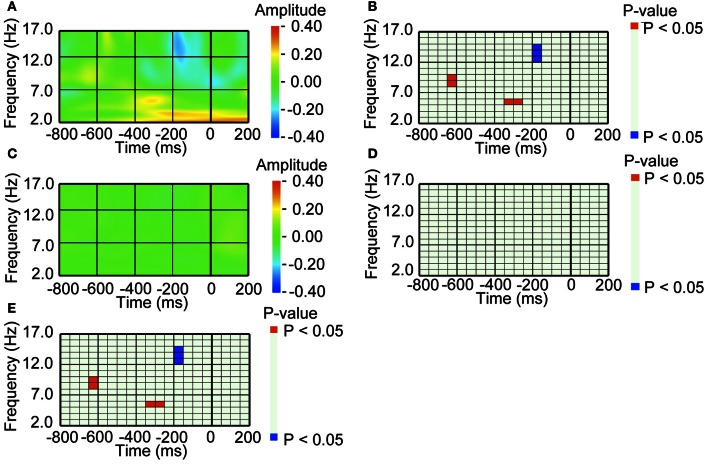
**EEG amplitude preceding a saccade, when a mother wavelet with a size of 3 cycles was used for the continuous wavelet transform. (A)** Average waveform of the occipital recordings (O2) aligned with the *pre-switch* saccades that were followed by perceptual switching. The amplitude was Z-transformed, and the amplitude *zero* indicates baseline amplitude. **(B)** Statistical difference between average waveform aligned with the *pre-switch* saccades and baseline amplitude. The colors *red* and *blue* denote that amplitude was larger in the average waveform or baseline amplitude, respectively (*P* < 0.05). **(C)** Average waveform of the occipital recordings (O2) aligned with the *no-switch* saccades. The amplitude was Z-transformed, and the amplitude *zero* indicates baseline amplitude. **(D)** Statistical difference between average waveform aligned with the *no-switch* saccades and baseline amplitude. The *colors red* and *blue* denote that amplitude was larger in average waveform or baseline amplitude, respectively (*P* < 0.05). **(E)** Statistical difference between the average waveform aligned with the *pre-switch* saccades and the average waveform aligned with the *no-switch* saccades. The colors *red* and *blue* denote that amplitude was larger in the *pre-switch* saccade-related average waveform or the *no-switch* saccade-related average waveform, respectively (*P* < 0.05).

The mother wavelet used in continuous wavelet transform of EEG had a width of 5 cycles. At 10 Hz, EEG in a width of 500, 250 ms before and 250 ms after, could affect amplitude at time of interest in time-frequency domain. To check whether amplitude in- or decrease before *pre-switch* oculomotor events were due to some part of oculomotor events bleeding in to the pre-oculomotor events activity, we applied the same analyses with a mother wavelet, of which the width was 3 cycles; we obtained similar results (see Figures A1, A2 of Appendix).

## Discussion

We analyzed the properties of EEG episodes preceding the onset of eye-blinks and saccades. Occipital alpha band activity prior to blinks and saccades was predictive of whether these would lead to subsequent switching of perceived orientation in the Necker cube. When the occipital alpha band activity exhibited increased amplitude, blinks led to perceptual switching, and when it exhibited an increase followed by a decrease in amplitude, the saccades led to perceptual switching.

Alpha band activity at occipital sites has been associated with attentional deployment. For example, an anticipatory shift of visual attention to a target decreases the amplitude of alpha band activity in cortical areas tuned to the newly attended location (Sauseng et al., 2005; Yamagishi et al., 2005, 2008; Thut et al., 2006; Rihs et al., 2009), suggesting that a decrease in alpha reflects facilitation of future visual processing. On the other hand, cortical areas that are tuned to unattended locations exhibited increased alpha amplitude (Worden et al., 2000; Sauseng et al., 2005; Kelly et al., 2006; Rihs et al., 2007), suggesting that this reflects active inhibition of task-irrelevant processing (Klimesch et al., 1999, 2007). Thus, a sequence of increased and decreased alpha band activity would contribute to the deployment of visual spatial attention through disengaging and shifting of attention, respectively.

Attentional deployment might facilitate perceptual switching. According to the focal-feature hypothesis (Toppino, 2003), different focal regions within an ambiguous figure favor one perception of an ambiguous figure over another, by selectively enhancing a certain part within the visual input (Chelazzi et al., 2001).

Both blinks and saccades are associated with attentional process. Blinkings tend to occur at breakpoints of attention (Nakano et al., 2009; Nakano and Kitazawa, 2010) and are involved in the process of attentional disengagement (Nakano et al., 2013). Thus, the combination of amplitude increase of the alpha band activity and blinking would reflect the disengagement of attention to initiate the process of perceptual switching. On the other hand, saccades are associated with the reallocation of spatial attention (Filali-Sadouk et al., 2010). The combination of amplitude decrease of the alpha band activity and saccade might reflect the shift of attention that elicits the process of perceptual switching.

Our observations were correlational and therefore we cannot point out a causal relationship between occipital alpha band activity and perceptual switching. It is possible that the alpha band activity appeared time-locked to blinks or saccades with no functional role in the switching process. Based on a number of studies about the alpha band activity and attentional deployment (Worden et al., 2000; Sauseng et al., 2005; Yamagishi et al., 2005, 2008; Kelly et al., 2006; Thut et al., 2006; Rihs et al., 2007, 2009), we suggest, nevertheless, that the presently observed amplitude modulation of the alpha band activity was associated with attentional processes that play an active role in perceptual switching.

In our previous study (Nakatani et al., 2011), we discussed possible relationships between *pre-switch* oculomotor events and posterior theta band (around 5 Hz) activity. The *pre-switch* blinks occurred 1000 ms prior to switching responses. The theta band activity appeared 400 ms after the blinks. *Pre-switch* saccades occurred 150 ms prior to switching responses. Here, the theta band activity appeared 400 ms before the saccades. The posterior theta band activity was also observed in the control condition, where presented stimuli were switched from one to other. As the theta band activity followed changes of presented stimuli, we considered it to reflect the change of percept. Taken together current findings and previous findings, we may describe the processes of perceptual switching as follows.

In the case of *pre-switch* blinking, first a disengagement of attention occurs, reflected by the amplitude increase of the alpha band activity. The subsequent blink facilitates the detachment of attention from a part of the Necker cube that corresponds to the current percept. This process is more likely to be observed during the non-preferred interpretation of the Necker cube, as blinks tend to be followed by a switch to the preferred interpretation. Saccades that occur between the amplitude increase of the alpha band activity and the *pre-switch* blinks likewise facilitate detachment of attention by shifting the gaze from an attended location to other location. After attentional disengagement, the process of changing the percept is reflected by the theta band activity. In the case of the *pre-switch* saccade, first a disengagement of attention occurs, reflected by amplitude increase of the alpha band activity 600 ms before saccades. Second, the process of changing the percept is reflected by the theta band activity. Then, the shift of attention occurs to facilitate the change of percept, reflected by amplitude decrease of the alpha band activity and saccade whose direction was associated with the interpretation of the Necker cube after the switch (Nakatani et al., 2011), and it leads to perceptual switching.

In conclusion, we pointed out that preceding occipital alpha band activity predicts the impact of oculomotor events on current percept during perception of an ambiguous figure. Our results suggest that spontaneous oculomotor events dynamically play an active role in perceptual organization.

### Conflict of interest statement

The authors declare that the research was conducted in the absence of any commercial or financial relationships that could be construed as a potential conflict of interest.

## Appendix

The mother wavelet used in the continuous wavelet transform of EEG had a width of 5 cycles. At 10 Hz, oculomotor behavior in a width of 500, 250 ms before and 250 ms after, could affect amplitude of EEG at the time of interest. To check whether amplitude increase or decrease before *pre-switch* oculomotor events were due to some part of oculomotor event-related activity bleeding in to the pre-oculomotor events activity, we applied the same analyses with a mother wavelet of which the width was 3 cycles. Figure A1 is for amplitude of EEG prior to blinks, and Figure A2 is for amplitude of EEG prior to saccades. The results are similar to those obtained with a 5-cycles wavelet analysis.
